# Editorial: Multiple Roles of Alien Plants in Aquatic Ecosystems: From Processes to Modelling

**DOI:** 10.3389/fpls.2020.01299

**Published:** 2020-08-21

**Authors:** Rossano Bolpagni, Lorenzo Lastrucci, Giuseppe Brundu, Andreas Hussner

**Affiliations:** ^1^ Department of Chemistry, Life Sciences and Environmental Sustainability, Parma University, Parma, Italy; ^2^ Natural History Museum of the University of Florence – Botany, Florence, Italy; ^3^ Department of Agriculture, University of Sassari, Sassari, Italy; ^4^ Department of Ecosystem Research, Leibniz-Institute of Freshwater Ecology and Inland Fisheries, Berlin, Germany

**Keywords:** non-native macrophytes, biological invasions, ecosystem processes, biotic-abiotic interactions, aquatic ecosystems

## Background

Invasive alien species are one of the most significant contemporary challenges threating biodiversity, ecosystem functioning, and human wellbeing (Rai and Singh, 2020), resulting in major economic and environmental damages and losses (Pimentel et al., 2005). Biological invasions are favored by ecosystems’ over-exploitation and climate change, and the progressive accumulation of invasive species strongly weakens the invaded communities by occupying empty phylogenetic and functional spaces or by excluding natives (Dalle Fratte et al., 2019). Among ecosystems, freshwaters seem to be particularly prone to invasions due to their high natural dynamism associated with a global hydrological and trophic alteration due to human activities (Dudgeon, 2019). This urgently calls for a better understanding of the multiple roles played by invasive aquatic alien plants (IAAPs). To this regard, the present Research Topic offers novel perspectives on IAAPs science and on the implications of their establishment.

## Environmental Drivers and Functional Traits of IAAPs

A key question is how IAAPs can progressively replace native counterparts. The invasive success of IAAPs depends in part on the characters of invaded ecosystems, as highlighted by Shen et al. Testing the role of sediments in enhancing the invasibility of aquatic ecosystems, they showed that the soil spatial heterogeneity might have a primary role in promoting the invasive success of *Myriophyllum aquaticum* by regulating morphological traits of individual clonal ramets. Wu and Ding reviewed the role of fundamental drivers such as global change, water pollution, and economic growth in IAAPs introduction and invasions in China, clarifying that “support to aquaculture” and “ecological restoration” are major introduction pathways. Furthermore, IAAPs invasion risk is expected to be boosted by climate change, as well as by increased nutrient availability, and it seems to be under the total control of future economic trends.

Beside the invasibility of the ecosystems, the attributes of IAAPs play a major role for successful invasion. Szabó et al. explored the eco-physiology of two invasive elodeids (*Elodea canadensis* and *E. nuttallii*) as a key to understanding their relative invasiveness. Their investigation of the combined effects of nutrient and light environment demonstrated that relative growth rate, chlorophyll concentration, and efficiency of photosystem II provide *E. nuttalli* the ability to replace congeneric species in the invaded sites. Similarly, Tóth et al. discussed the roles of physiological and ecological traits in explaining the differentiated performances of invasive macrophytes, obtaining evidence on the existence of key adaptive responses of IAAPs. Importantly, *Ludwigia hexapetala*—one of the most invasive IAAPs globally—showed exceptionally high photosynthetic efficiency, resulting in an elevated tolerance to high light intensities. Both papers reinforce the direct implication of plant traits for the invasiveness of IAAPs, suggesting the importance to gain knowledge on this topic. In this respect, Asaeda et al. have investigated habitat preference of *Egeria densa*, focusing on physiological stress proxies. Based on the tissue concentrations of hydrogen peroxide (H_2_O_2_), it is possible to define critical thresholds for the species survival in terms of flow velocity and light intensity, with relevant implications for designing management solutions to counteract the spread of IAAPs.

## IAAPs and Biological Interactions

However, beyond the characteristics of habitats and IAAPs, biological interactions—including competition, facilitation, invasional meltdown, and novel weapons—are also crucial for enlightening the IAAPs success. The study by Michelan et al. outlined how the influence of IAAPs on the colonization performance of native species is density dependent, studying the alien *Urochloa arrecta* and the two native *Pontederia cordata* and *Leersia hexandra* performances in terms of biomass and plant length. In their manipulative experiment, they found that if *U. arrecta* density increases (as number of fragments), the biomass and length of native species decrease substantially. However, the responses of the native species to the invader is species-specific, with differentiated biomass allocation behaviours. Wegner et al. investigated the biological mechanisms underlying the boom-bust dynamics of *E. nuttallii*, by modelling the temporal changes in water quality, and abundances of *E. nuttallii* and of another critical invader, the quagga mussel, in a temperate shallow lake. They found that the dynamics of multi-invasion processes can be characterized by different, contrasting phases. After an initial mutual facilitation—as predicted by the invasional meltdown hypothesis—a strong space competition followed. This study offers a new mechanistic explanation of the typical boom-bust dynamics of invasive elodeids.

Likewise, Strange et al. modelled the key interactions involved in the replacing of a floating IAAP (*Pistia stratiotes*) by a submerged one (*E. densa*), evaluating the implications of top-down vs bottom-up processes mediated by biological control efforts and nutrient loading, respectively. Their main message highlighted the risks associated with the adoption of control and eradication actions without planning active recovery interventions, as this lack may create opportunity windows for secondary invasions. Moreover, Thiebaut et al. examined the allelopathic potential of four aquatic plants (including IAAPs) on *L. hexapetala*, monitoring its relative growth rate and physiological traits in response to the exposition to root and leaf leachates of *L. peploides*, *M. aquaticum*, and *Mentha aquatica*, finding complex allelopathic responses. Specifically, *L. hexapetala* exibits a positive autoallelopathy which explains its invasiveness. In addition, they highlighted the risk of occurrence of potential invasional meltdown phenomena, with huge consequences on the local success of *L. hexapetala*, and invasive *Ludwigia* species in general.

## IAAPs as Ecosystem Engineers

IAAPs can deeply alter the functioning of ecosystems. Ribaudo et al. stressed the biogeochemistry implications of *E. densa* and *Lagarosiphon major*, as they are capable to trigger huge stratification events for several key chemical parameters (O_2_, C, N), as recurrent hypoxia events. They confirmed that the spread of IAAPs involved a change in the metabolism of ecosystems, resulting in a net nutrient release towards the water column, concurrently with a net increase in carbon emission towards the atmosphere.

## Perspectives on IAAPs Science

This Research Topic goes beyond the classical simplistic paradigm on the only negative impacts of IAAPs and increases the awareness on the multiple roles played by IAAPs in aquatic ecosystems. To date, available knowledge is not sufficient for an effective control and management of aquatic invaders (Hussner et al., 2017; Hofstra et al., 2020). Additional global efforts are needed to organize in a comprehensive and cross-cutting framework the processes of introduction, establishment, and spread, as well as the assessment of ecosystem changes and impacts mediated by IAAPs, keeping an eye on genetic and functional aspects (Lastrucci et al., 2018; Molina-Montenegro et al., 2018). In this frame, all the papers collected here will boost scientific interest in these issues, reaffirming the importance of tailored researches to get satisfactory answers to complex issues.

## Author Contributions

All authors conceived the idea for the special issue. RB coordinated the reviewing and editing of the papers included in the special issue and wrote the first version of the Editorial with major inputs from LL, GB and AH. All authors commented on the Editorial, read, and approved the final version.

## Conflict of Interest

The authors declare that the research was conducted in the absence of any commercial or financial relationships that could be construed as a potential conflict of interest.
